# Uptake of Colorectal Cancer Screening Among Deaf Adults in the United States

**DOI:** 10.7759/cureus.95403

**Published:** 2025-10-25

**Authors:** Adeyinka Laiyemo, Lamiaa Rougui, Angesom Kibreab, Victor F Scott, Zaki Sherif, Hassan Brim, Hassan Ashktorab, Adedoyin Kalejaiye, Farshad Aduli, Shelly McDonald-Pinkett

**Affiliations:** 1 Internal Medicine, Howard University College of Medicine, Washington DC, USA; 2 Gastroenterology, Howard University College of Medicine, Washington DC, USA; 3 Biochemistry, Howard University College of Medicine, Washington DC, USA; 4 Pathology, Howard University College of Medicine, Washington DC, USA; 5 Surgery, Howard University College of Medicine, Washington DC, USA

**Keywords:** cancer prevention, cancer screening, colorectal cancer, deaf, hearing impaired

## Abstract

Background: People with disabilities face challenges in access and utilization of healthcare resources in part due to the additional resources needed for their healthcare delivery. There is little information about colorectal cancer (CRC) screening uptake among deaf and hearing-impaired US adults. We evaluated the uptake of CRC screening among deaf US adults.

Methods: We used the 2018 Health Information National Trends Survey (HINTS 5 Cycle 2). Our study cohort included 2,049 respondents (weighted population size = 107,282,358) without a personal history of CRC who were at least 50 years old, reported whether they were deaf or not, and answered questions regarding their ever use of CRC screening modalities (fecal immunochemical test, sigmoidoscopy, or colonoscopy). We used logistic regression analyses to examine the association of deafness with ever being screened for CRC. We used survey weights in all analyses. We calculated odds ratios (OR) and 95% confidence intervals (CI).

Results: There were 202 deaf respondents (weighted population size = 9,994,286) and 1,847 people with normal hearing (weighted population size = 97,288,072). Deaf respondents were older (mean age 70.6 years versus 62.1 years) and were more likely to be male (n = 116 (59.9%)) versus female (n = 86 (40.1%), P = 0.004). CRC screening uptake was similar among deaf and normal hearing adults (n = 173 (80%) versus n = 1,493 (72.6%), respectively; unadjusted OR = 1.51; 95% CI: 0.80-2.83 and adjusted OR = 0.71; 95% CI: 0.32-1.60).

Conclusion: Hearing impairment was not found to be a barrier to the uptake of CRC screening among US adults.

## Introduction

Colorectal cancer (CRC) is the second leading cause of cancer-related deaths in the United States [1]. Screening has been proven in randomized trials to reduce mortality from CRC [2-4]. However, screening for CRC is suboptimal in the United States in general [5,6] and is particularly poor among minority populations based on race-ethnicity and socioeconomic status [7-11].

It is well known that people with disabilities face challenges in healthcare access and utilization of healthcare resources in part due to the additional resources needed for their healthcare delivery [12]. Deaf people often require American Sign Language (ASL) interpreters for optimal communication with their care providers; otherwise, the consultation is unduly prolonged. The sad reality is that many times, ASL interpreters are not available during consultations, and web-based interpreter services that are compliant with established healthcare practices may not be readily available, hence the communication challenge of deaf people with optimal healthcare services uptake [13-15]. It has been shown that deaf patients lag in cervical cancer screening [16], are less likely to have information about appropriate lung cancer screening [17], and are less likely to be engaged in shared decisions with respect to prostate cancer screening [18]. There is a lack of information about CRC screening uptake among deaf people. However, it has been reported that when physicians communicate and recommend CRC screening to their patients, they are more likely to be screened for CRC [19]. In this study, we evaluated CRC screening among deaf adults in the United States.

An abstract from this study was presented in October 2021 at the American College of Gastroenterology scientific meeting in Las Vegas, Nevada, USA.

## Materials and methods

We used data from the Health Information National Trends Survey (HINTS), a nationally representative survey conducted by the National Cancer Institute (NCI). This survey asks questions about health-related information and the behavior of US adults. The 2018 HINTS 5 Cycle 2 data were conducted from January to May 2018 and collected data about the knowledge of, attitude toward, and use of cancer- and health-related information among Americans. The survey was conducted exclusively via mail, and toll-free phone numbers were provided to respondents to voice questions, concerns, or request materials in Spanish. More information about the HINTS 5 Cycle 2 survey and the deidentified dataset can be found on the official website of the NCI at https://hints.cancer.gov/ [20].

We obtained exempt approval from the Institutional Review Board of Howard University in Washington DC (IRB-14-MED-28) and downloaded the deidentified dataset. Our analytic cohort included 2,049 respondents (weighted population size = 107,282,358) without a personal history of CRC who were at least 50 years old, answered questions regarding their ever use of CRC screening modalities (fecal immunochemical test, sigmoidoscopy, or colonoscopy), and responded to the question “Are you deaf or do you have serious difficulty hearing?”. We classified participants with a “yes” response as “deaf” and those who responded with a “no” response as normal hearing.

We compared the demographic and lifestyle factors of deaf respondents and normal hearing respondents. We analyzed age as a continuous variable and compared the mean ages of deaf and normal hearing respondents and reported the 95% confidence intervals (CI). We compared men to women and categorized race as White, Black, Hispanic, and others for our analysis. We also categorized the highest education attainment as high school or less versus some college/vocational training versus college degree holders. We analyzed smoking history as never smokers versus former smokers versus current smokers. We analyzed body mass index (BMI) as normal versus overweight versus obese. All categorical variables were compared using a chi-squared test with survey weights using "svy tab" function in Stata statistical software (StataCorp LLC, College Station, TX, US). The corresponding P values were reported, with values less than 0.05 being regarded as statistically significant. We used logistic regression analyses to examine the association between deafness and the likelihood of ever undergoing CRC screening. We used survey weights in all analyses. We calculated odds ratios (OR) and 95% CI. Our final model included age, sex, smoking, health insurance, race-ethnicity, BMI, and highest education attained.

## Results

A total of 202 respondents were deaf (weighted population size = 9,994,286), and 1,847 people reported normal hearing (weighted population size = 97,288,072). Deaf respondents were older (mean age 70.6 years versus 62.1 years). Deaf respondents had male preponderance (59.9% versus 40.1%, P = 0.004) and had less formal education (high school or less: 50.5% versus 35%, P = 0.004). There were no differences by race-ethnicity, marital status, smoking history, degree of obesity, and health insurance (Table 1).

**Table 1:** Comparison of characteristics of respondents with and without deafness All percentages are weighted CI: confidence interval

Characteristics	Normal hearing, N = 1,847 (90.7%)	Deaf, N = 202 (9.3%)	P value
Mean age (years) (95% CI)	62.1 (61.5-62.7)	70.6 (68.5-72.7)	
Sex, n (%)			0.004
Male	765 (46.2)	116 (59.9)	
Female	1,082 (53.8)	86 (40.1)	
Race, n (%)			0.72
White	1,142 (70.8)	131 (74.3)	
Black	253 (11.5)	9 (7.6)	
Hispanic	199 (12.2)	27 (12.8)	
Other	108 (5.5)	12 (5.3)	
Education status, n (%)			0.004
High school or less	518 (35)	83 (50.5)	
Some college/vocation	576 (38.9)	61 (34.0)	
College graduate	745 (26.1)	56 (15.5)	
Marital status, n (%)			0.26
Unmarried	907 (40.6)	95 (35.4)	
Married	928 (59.4)	106 (64.6)	
Insurance status, n (%)			0.91
Uninsured	65 (5.7)	6 (5.3)	
Insured	1,757 (94.3)	190 (94.7)	
Smoking status, n (%)			0.06
Never	1,061 (55.7)	108 (53.1)	
Former	525 (27.7)	68 (36.7)	
Current	237 (16.6)	23 (10.3)	
Body mass index (kg/m^2^)			0.21
<25	545 (29.8)	43 (21.5)	
25-29	663 (36.7)	77 (37.4)	
≥30	594 (33.5)	73 (41.1)	

Among deaf people, 80% reported ever being screened for CRC while 72.6% of normal hearing respondents have ever been screened. In the unadjusted model, there were no significant differences in CRC screening uptake among deaf respondents when compared to normal hearing respondents: OR = 1.51; 95% CI: 0.80-2.83. The addition of age to the model reversed the OR to 0.91. After adjustments for additional a priori factors associated with CRC screening uptake, ever screened for CRC was comparable among deaf and normal hearing adults in the United States: OR = 0.71; 95% CI: 0.32-1.60 (Table 2).

**Table 2:** Association of deafness with CRC screening among US adults Multivariate model adjusted for age, sex, race-ethnicity, insurance, marital status, BMI, and smoking CRC: colorectal cancer; OR: odds ratio; CI: confidence interval; BMI: body mass index

Characteristics	CRC screening uptake					
Deaf	Had CRC screening		Unadjusted		Adjusted	
	n	Weighted %	OR	95% CI	OR	95% CI
No (N = 1,847)	1,493	72.6	Reference	Reference	Reference	Reference
Yes (N = 202)	173	80	1.51	0.80-2.83	0.71	0.32-1.60

## Discussion

In this study, we used the HINTS 5 Cycle 2 to evaluate the uptake of CRC screening among deaf people when compared to normal hearing people in the United States. We found comparable uptake among deaf and normal hearing persons, indicating that deafness as a disability did not affect CRC screening.

We are not aware of any previous study that has evaluated CRC screening among deaf people for a direct comparison to our study. However, Kushalnagar et al. [16] evaluated cervical and breast cancer screening among deaf and normal hearing women in the United States. The authors reported that deaf people were less likely to be screened for cervical cancer: 78% versus 85%; OR = 0.71; 95% CI: 0.59-0.86; P < 0.001. However, deaf people had a comparable rate of breast cancer screening: 76% versus 82%; OR = 0.94; 95% CI: 0.77-1.15 [16]. While the reason behind the comparable CRC screening uptake observed in our study is unknown, we speculate that increasing awareness of the benefit of CRC screening may be a positive driving factor in this regard.

It is important to establish optimal communication between care providers and their patients, especially in the delivery of preventive healthcare services such as CRC screening. We have previously reported that patients were more likely to be compliant with CRC screening guidelines after they have had discussions with their care providers about screening (OR = 8.83; 95% CI: 7.20-10.84) and were more likely to be up to date when their care providers made specific recommendations about the screening modality rather than leaving it to the patient to decide (OR = 2.04; 95% CI: 1.54-2.68) [19]. In the present study, we found that deaf patients in the United States have comparable CRC screening as normal hearing Americans. This is a positive finding that the direct communication limitation of deaf people did not negatively impact CRC screening uptake.

A notable strength of the present study is that we studied a large, nationally representative sample of US adults, and we evaluated a segment of the population that is not often studied. However, our study has some limitations too. Our findings were based on self-reports in a cross-sectional survey, and the study was a secondary analysis of a deidentified sample. Therefore, we could not contact the respondents nor review their medical records for verification. Furthermore, we did not have any information about the severity of the hearing loss or the participants’ preferred mode of communication, whether verbal, ASL, or written.

## Conclusions

In this study, our findings suggest comparable CRC screening uptake among deaf and hearing adults in this dataset; however, further research is warranted to confirm these results in larger and more detailed cohorts. Nonetheless, the implication of our finding is that it is reassuring that we did not find a detectable difference in CRC screening uptake among adults with or without hearing impairments in the United States. The additional resources that persons with disability require for optimal utilization of healthcare resources should continue to be provided in all healthcare systems. However, there is a continuing need to increase CRC screening among all segments of the population in order to ensure that "no colon is left behind."
